# Chronic non-specific low back pain – sub-groups or a single mechanism?

**DOI:** 10.1186/1471-2474-9-11

**Published:** 2008-01-25

**Authors:** Benedict Martin Wand, Neil Edward O'Connell

**Affiliations:** 1 School of Health Sciences, University of Notre Dame, Australia, 19 Mouat St, Fremantle WA 6959, Australia; 2 Centre for Research in Rehabilitation, School of Health Sciences and Social Care, Brunel University, Uxbridge, Middlesex UB8 3PH, UK

## Abstract

**Background:**

Low back pain is a substantial health problem and has subsequently attracted a considerable amount of research. Clinical trials evaluating the efficacy of a variety of interventions for chronic non-specific low back pain indicate limited effectiveness for most commonly applied interventions and approaches.

**Discussion:**

Many clinicians challenge the results of clinical trials as they feel that this lack of effectiveness is at odds with their clinical experience of managing patients with back pain. A common explanation for this discrepancy is the perceived heterogeneity of patients with chronic non-specific low back pain. It is felt that the effects of treatment may be diluted by the application of a single intervention to a complex, heterogeneous group with diverse treatment needs. This argument presupposes that current treatment is effective when applied to the correct patient.

An alternative perspective is that the clinical trials are correct and current treatments have limited efficacy. Preoccupation with sub-grouping may stifle engagement with this view and it is important that the sub-grouping paradigm is closely examined. This paper argues that there are numerous problems with the sub-grouping approach and that it may not be an important reason for the disappointing results of clinical trials. We propose instead that current treatment may be ineffective because it has been misdirected. Recent evidence that demonstrates changes within the brain in chronic low back pain sufferers raises the possibility that persistent back pain may be a problem of cortical reorganisation and degeneration. This perspective offers interesting insights into the chronic low back pain experience and suggests alternative models of intervention.

**Summary:**

The disappointing results of clinical research are commonly explained by the failure of researchers to adequately attend to sub-grouping of the chronic non-specific low back pain population. Alternatively, current approaches may be ineffective and clinicians and researchers may need to radically rethink the nature of the problem and how it should best be managed.

## Background

Low back pain (LBP) is a substantial health problem. It affects up to 80% of the adult population [1] and accounts for considerable healthcare and socioeconomic costs [2]. International guidelines for the management of LBP recommend an initial triage to facilitate effective management of the problem [3]. This classification process differentiates between specific spinal pathology, nerve root pain and simple or non-specific low back pain (NSLBP) [4]. Most authorities suggest further staging the problem by symptom duration into acute, sub acute or chronic [5-7]. NSLBP represents about 85% of LBP patients seen in primary care [8] and the vast majority of LBP patients seen by physical therapists are classified under this label.

The prognosis for acute NSLBP (ANSLBP) is relatively favourable. A significant percentage of sufferers, probably over 50%, do not consult a health care professional for the problem [9]. Among those who do seek care, most will experience rapid improvement in pain and disability within the first three months [10]. Beyond this time the majority no longer consult and will continue to experience only low levels of pain and disability, and most have returned to work and their usual daily activities [10-12].

In a small group of acute patients, the problem fails to resolve as it should. Perhaps 10% will go on to develop chronic, disabling LBP [12-14]. It is this group that utilises the majority of resources allocated [11] and subsequently there has been a considerable research effort to develop and evaluate effective treatments for this group.

Recent systematic reviews of the most commonly applied treatments reach remarkably similar conclusions. Most treatments provide small, short-term benefits when compared to no treatment or sham treatment, but offer little benefit when compared to other forms of intervention [15]. No treatment seems to be superior to any other intervention, including usual GP care [7,15-17]. Furthermore, none of the cited interventions can be truly said to offer a solution to the problem of chronic NSLBP (CNSLBP). Although the magnitude of an individual's problem may be reduced, the reduction is typically small and the problem still persists [7,17,18].

Recent work evaluating the clinical importance of outcome in CNSLBP reinforces this perspective. Keller et al [19] calculated the effect sizes for a number of conservative interventions when compared to a no treatment control group. Acupuncture, exercise and psychological treatment demonstrated moderate effect sizes for short-term pain relief. Small effect sizes were seen for manual therapy and electrical stimulation. Effect sizes for short-term changes in function were small for exercise and psychological treatment. Long-term data were only presented for exercise, which demonstrated small effect sizes for both pain and function. They concluded that there is a dire need to develop more effective interventions for CNSLBP.

van Tulder et al. [20] evaluated the effect size of exercise based treatments for CNSLBP by relating the between group difference in outcome to the minimal clinically important difference (MCID) for that outcome. Of 37 trials analysed, only seven showed a clinically important improvement in outcome. The same group performed a meta-regression on trials of exercise for CNSLBP in an attempt to determine the characteristics of exercise that may optimise outcome from this intervention [21]. A multivariate model including the most effective intervention characteristics had only a 4% chance of producing clinically meaningful change in function when compared to no treatment and a 1% chance of producing meaningful improvement in function when compared to other conservative treatment. There was a 29% chance that the expected improvements in pain would be clinically meaningful in comparison to no treatment and a 3% chance when compared to other active treatment.

The disappointing results from clinical trials may be interpreted in two ways. Firstly, it is possible that the clinical trials are wrong. That is, for various reasons, the clinical trial process fails to capture the reality of the clinical setting and underestimates the true effectiveness of current practice. Alternatively, the clinical trials are right and current approaches to the management of CNSLBP are of limited value.

The former perspective is one commonly taken by clinicians who feel the results of clinical trials are at odds with their clinical experience. Numerous explanations have been proffered, including insufficient treatment dose [22], inappropriate patient selection [23,24], unsuitable treatment protocols [25] and outmoded treatment [26]. Probably the most commonly encountered explanation relates to the perceived heterogeneity of the CNSLBP population and the failure of research to adequately account for this heterogeneity. Many authors feel that CNSLBP is made up of several distinct sub-groups, each reflecting different mechanisms of symptom production [27-29]. It is argued that the effects of treatment are diluted by the application of a single intervention to a complex, heterogeneous group with diverse treatment needs [28-33]. The consequence of this assertion is that sub-groups within the CNSLBP population need to be identified to enable matching of the intervention with each specific mechanism [28,29,31,32]. This approach has within it the assumption that current interventions are appropriate and will ultimately be shown to be successful, if only they can be applied to the correct patient.

The alternative interpretation, that the clinical trials are correct and current practice offers little benefit, is often neglected, and along with it the research agenda that would have as its priority the development of new and novel models of care for CNSLBP. The purpose of this paper is to encourage clinicians to consider this alternative. Particularly, that for management to move forward clinicians may need to regard CNSLBP in a very different way. We will attempt to do this in two ways. Firstly, we feel that the sub-grouping paradigm potentially stifles engagement with this view and believe it is important that clinicians and researchers more closely scrutinise the sub-grouping approach. Accordingly, the first section of the discussion attempts to do this by considering possible weaknesses in the sub-grouping perspective. Secondly, an alternative model for understanding CNSLBP will be presented, which offers potential for very different ways of managing the problem

## Discussion

### Sub-grouping is only one of a number of possible explanations for the manifestations of CNSLBP

What clinicians and researchers mean by a sub-group is not always clear. Sometimes it may refer to clustering of effect modifiers within a distinct diagnostic group, what may be referred to as a 'clinical sub-group'. Other times the term is used to indicate groups with a unique mechanism or cause underlying the disorder, the terms 'aetiological' or 'mechanistic sub-group' probably best capture this perspective. Both are seen in the LBP literature, for example the three major categories in the McKenzie approach [34] can be thought of as mechanistic sub-groups, whereas the sub-classifications of 'derangement' by directional preference and symptom distribution represent clinical sub-groups under this single mechanistic umbrella. Likewise, O'Sullivan [35] has proposed three broad mechanistic sub-groups for CLBP. These have similarly been divided into clinical sub-groups; the broad aims of treatment are the same for each mechanistic sub-group though the specifics of intervention vary within the clinical sub-classifications. It is mechanistic sub-grouping that is under discussion in this paper. Patients' presentations and responses to treatment will always vary and require personalisation of care. However, it is important to consider if these variations need be interpreted as indicating distinctly different mechanisms underlying the disorder.

The statistical problems and pitfalls of sub-grouping have been reviewed extensively in the literature [36-38]. However, other forms of bias in the construction of mechanistic sub-groups have been less often discussed. In particular, it is possible that a form of confirmatory bias pervades aspects of the sub-grouping argument [39]. That is, explanations for the phenomena of LBP other than sub-grouping are not considered. There are a number of characteristics of the LBP experience that have prompted the proposition of a sub-grouping approach, and while sub-grouping is consistent with these, it is worthwhile to consider other potential explanations.

There is no doubt that CNSLBP represents a complex, multi-dimensional problem. The chronic LBP experience is characterised by a vast array of physical [40-42] and psychosocial [43,44] features. It is this complexity that is cited as a compelling argument for the existence of distinct mechanistic sub-groups within the CNSLBP population [31,45]. Complexity in presentation does not have to be interpreted as evidence of distinct mechanisms. The literature is replete with descriptions of the same degree of physical and psychological intricacy within discrete diseases [46-52]. In these examples, the presence of a single disease gives rise to a host of physical and psychological characteristics. As with LBP, these characteristics contribute to the consequences of the problem to that individual. The understanding of this complexity is vital in helping personalise treatment and determining best how treatment goals may be obtained for each patient. However, there is a distinction between varying and personalising individual care within a single framework and seeking distinctly different modes of care.

The multi-dimensional nature of CNSLBP may also be interpreted in this way. While complexity in behaviour can be explained by distinct mechanistic sub-groups, it can equally be interpreted as the individualisation of response to a single underlying problem, and while the complexity of the presentation requires that intervention be broad and personalised it may still have as its basis a single idea.

Not only is CNSLBP complex, it is also possible to observe clustering of various characteristics in this population [31,33,53,54]. Historically, diagnosis has been driven by labelling clusters of signs and symptoms [55] and in CNSLBP it may be that these clusters represent different mechanisms requiring different interventions [28]. However, it is again worth considering other explanations. Researchers have repeatedly demonstrated grouping of characteristics in patients with specific disease processes. For example, patients with Parkinson's disease may be stratified by rate of clinical progression [56] and individuals with sickle cell disease can be grouped by level of fear avoidance [57]. Clustering of signs and symptoms will almost always be possible within single disease entities. One reason for this is variability in the severity of the condition. It is a reasonable prediction that those patients whose condition is less severe will exhibit different signs, symptoms and behaviours to those whose condition is moderate or those whose condition is severe. Clustering in presentation does not necessarily represent evidence of separate mechanisms. Different mechanisms may explain clustering, but equally it is possible to see and explain this phenomenon in problems with a single aetiology.

Another consideration is the variability seen in clinical trials of treatment for CNSLBP. Most commonly applied interventions have been subject to numerous studies, and for any given intervention it is possible to identify some studies in which there is a demonstrated effect and others in which there is no effect. Failure to account for sub-groups within the LBP population has again been cited as a potential explanation for these findings [38,32].

There are several alternative explanations that are worthwhile contemplating. Firstly, statistical power may underpin some of the variability seen. The inconsistency does not necessarily reflect variability in treatment efficacy, just variation in the statistical efficiency of studies to detect treatment effects. Secondly, there is likely to be some random error associated with a large number of studies. One would expect to see most studies yield results representative of the average effect of that intervention. Likewise, there would be some variability in the response, with a few studies showing effects greater than the average and a few studies showing effects lower than the average. The variation may simply be noise, rather than variability induced by "innovative trial design" [32]. Finally there is some suggestion that authorship may influence the interpretation of results. Variability in the outcome of clinical trials may simply reflect different interpretations of effect sizes by authors [58,59], rather than evidence of bias in trial design or greater attention to sub-grouping.

A further commonly applied argument for the pursuit of mechanistic sub-groups is that NSLBP is too broad a category. Researchers suggest that since the majority of low back pain patients fall into this category, it's value in characterising a patient's presentation is only marginally more helpful than simply stating that they have pain in the back [31,40]. NSLBP is indeed a broad category, representing the vast majority of those who develop a complaint of back pain. However, CNSLBP represents only a small percentage of this population, probably less than 10% [12-14]. It is a highly stratified group, representing that collection of patients whose symptoms were severe enough to warrant consultation, who had neither specific spinal pathology nor nerve root pain and whose symptoms failed to settle in the usual time frame.

This section concludes that a number of the features of CNSLBP can be explained by mechanisms other than the inculcation of mechanistic sub-groups. This does not invalidate sub-grouping, it merely recognises that it is one of several alternatives. A more robust critique of the sub-grouping perspective requires consideration of the predictions that a sub-grouping model makes, and investigation of data related to these predictions. This is the aim of the next section.

### Clinical studies on CNSLBP do not strongly support the presence of mechanistic sub-groups

Supporters of the sub-grouping model contend that the small effect sizes seen in clinical trials result from the large positive effects seen in some patients being 'washed out' by the smaller or negative effects seen in others [32]. The solution offered for this problem is the matching of a diagnostic profile with the type of intervention best suited to that profile [32]. If this is the case there are a number of reasonable predictions that can be made from the clinical literature. Firstly, it would be expected that a treatment process that is derived from a patient examination and tailored to the individual's presentation would be superior to a treatment process that is not. Secondly, multimodal care is likely to be superior to unimodal care as there is less chance of 'washout' of treatment effect. Thirdly it should be possible to identify groups of patients who respond favourably to one form of treatment and less favourably to alternative treatments.

### Specific versus non-specific treatments

Several different protocols have been used that compare tailored to non-tailored treatment programmes, and all reach similar conclusions. Comparisons between treatment programmes based on individual patient assessment and more general and generic treatment programmes demonstrate equivalence in outcome [60-68]. Providing therapists with the opportunity to assess patients and provide them with the care they consider best addresses the mechanism underpinning their disorder does not seem to be better than treatment that is generically applied. Patients participating in general physical activity appear to do better than those engaged in specific back exercises [69] and level of training and experience of the therapist, presumably allowing more appropriate matching of treatment with the patients' profile, seems not to impact on outcome [70]. Overall, there seems to be little support for the view that specific, individualised treatment produces better outcome.

Kent and colleagues [71] systematically evaluated this perspective for manual therapy using meta-analysis. Their review compared the effects of manual therapy when therapists were free to select the intervention used, to studies in which the manual therapy treatment was prescribed. They were unable to detect any difference in outcome between these two conditions. Using a robust methodology, this group found no support for the perspective that individualised and clinically reasoned interventions are superior to generically applied interventions.

Hayden et al [21] compared outcome from individually tailored exercise programmes, which presumably sought to address the mechanisms underlying a patient's disorder, to standardised programmes. Individually designed programmes had no effect on function in comparison to general programmes and only a small and clinically unimportant influence on pain.

### Unimodal versus multimodal care

If the CNSLBP population contains distinct mechanistic sub-groups requiring different intervention strategies, then a reasonable prediction would be that multimodal interventions demonstrate superior outcomes to unimodal care, particularly when the unimodal components have been shown to impart some benefit to patients. The sub-grouping model would predict that subjects in multimodal care have a greater likelihood of receiving their appropriate intervention, thus decreasing the chance of 'washout'. While there is a possibility that treatments may interact negatively, or in other complex ways, we consider this a reasonable prediction. While some papers show small differences with the addition of treatments [72-74], the majority of studies report no difference when unimodal and multimodal approaches are compared [68,75-84]. Increasing the likelihood that patients are exposed to the 'correct' treatment does not seem to enhance outcome.

Hayden et al [21] again provide systematic and methodologically rigorous information on this perspective. They compared exercise only groups with groups that received exercise and additional therapy. The addition of other conservative care to exercise therapy for CLBP patients produced only small and clinically insignificant changes in pain and function.

### Sub-group response to treatment

One approach to investigating sub-grouping in LBP is to consider which cluster of patient factors identify those most likely to benefit from a particular treatment. Research on ALBP, for example, has identified distinct and theoretically plausible clinical profiles that predict success from manipulation [85] and stability training [86] and preliminary evidence suggests that grouping patients this way makes a significant difference to outcome [87,88].

If CNSLBP is similarly made up of different mechanistic subgroups, and a particular intervention is applied to the whole group, those patients that are matched to that treatment should obtain benefit and their clinical profile ought to emerge from analyses of treatment moderators. There have been many attempts to elucidate the features that influence outcome in CNSLBP patients receiving care. These data are difficult to interpret. The results are influenced by the type of outcome investigated and the follow-up interval [89]. In addition, there is great variability in the potential predictors that have been investigated, making comparison and synthesis difficult [90] and interpretation is often confounded by lack of a comparison group [91].

While acknowledging these problems, there is little evidence from clinical studies of a patient profile that responds uniquely to a given intervention. Pain intensity, duration of the problem and distress appear repeatedly as important predictors of outcome regardless of the type of treatment [30,90,92-96]. There is a sense that the characteristics of patients who do poorly or well with treatment are the same regardless of the intervention [92,93] and may simply be a reflection of severity. Unlike the situation in ALBP [85,86,97] it has not yet been demonstrated that the chronic population contains unique groups that respond differently to different interventions.

### What else may explain the disappointing treatment results?

In the previous sections we saw that the features of CNSLBP that have prompted clinicians and researchers to consider sub-grouping may have alternate explanations and that clinical data on CNSLBP does not strongly support the presence of sub-groups. Despite its popularity, sub-grouping may not be an important explanation for the disappointing results seen in clinical trials. An alternative perspective is that treatment has been ineffective because it has been misdirected. One possible scenario is that the problem of CNSLBP does not lie within the back, but within the brain. Thinking of CNSLBP as a problem of cortical reorganisation and degeneration may increase our understanding of the problem and more appropriately direct intervention.

There is growing evidence that the brains of patients with CNSLBP are different to those of normal subjects [98-102] and there are corresponding alterations in brain function [99,103,104]. Herta Flor's group [99] provided the initial data in this area. They recorded evoked magnetic fields in the brain in response to electrical stimulation of the back. They demonstrated greater reactivity in the primary somatosensory cortex (S1) and an expansion of the S1 representation of the back in CLBP patients. Altered S1 representation is also a feature of other complex pain problems [105,106].

Recently investigators have looked more widely at brain structure and function, and a consistent pattern of brain changes has begun to emerge. Siddall et al. [101] used magnetic resonance spectroscopy (MRS) to monitor biochemical changes in the anterior cingulate cortex, prefrontal cortex and thalamus of 32 CLBP patients. This method was highly accurate in distinguishing CLBP patients from normal volunteers. Grachev et al. [100] also studied the biochemical profile of these three brain regions. They looked at the concentrations of nine different metabolites using proton MRS. Again there was strong evidence for alterations in the biochemical profile of CLBP patients, most notable was evidence of neurodegeneration within the dorsolateral prefrontal cortex (DLPFC), a result replicated more recently in a group of depressed CLBP patients [107].

Brain morphometry has been used to investigate both general and regional gray matter density changes in CLBP patients. Apkarian et al. [98] reported a greatly decreased neocortical gray matter volume in patients compared to matched controls. Regional analysis supported the biochemical data, with the DLPFC showing the greatest extent of loss. Less extensive gray matter loss was also noted in the thalamus. Schmidt-Wilke et al. [102] also found gray matter loss in the DLPFC of CLBP patients as well as decreased volumes in S1 and the brainstem. This group also reported small increases in density in the thalamus and basal ganglia. Finally Honda et al. [108] looked at cerebral blood flow in CLBP patients as part of a larger study on diverse chronic pain states. Again the DLPFC featured, demonstrating decreased blood flow at rest. Decreased blood flow was also observed in the orbitofrontal cortex and anterior cingulate cortex.

Alteration in brain function may be the mechanism that underpins the problem of CNSLBP. The patterns of central changes are consistent across a number of studies and methodologies and the extent of the central nervous systems changes are proportional to the duration and severity of the condition [98,99,102]. Indeed, Baliki et al. [109] suggest that brain changes account for over 70–80% of the variance for intensity and duration of CLBP. Cross-sectional studies always raise the problem of causality, and while there is no equivalent information on CNSLBP, some small longitudinal studies on other pain problems characterised by altered cortical function suggest that interventions directed at cortical processing may be effective [110-113], in two studies more effective than conventional care [111,113]. Furthermore, good outcome is strongly related to normalisation of cortical function [114,115].

A cortical model of CNSLBP must be able to explain the complex clinical presentation of the problem. Critical challenges include accounting for the innumerable physical and psychological manifestations of CNSLBP and offering an explanation for the equivalence in outcome of very disparate treatments. Though speculative, alteration in brain function may offer some interesting perspectives and insights into these issues.

### Pain in the absence of meaningful spinal pathology

There is now strong evidence that structural changes within the spine have little meaning in the context of CNSLBP [18,116,117]. Recent evidence suggests that structural abnormalities have little impact on the outcome of conservative treatment [118] and invasive interventions, specifically designed to address putative peripheral pathology, appear to offer little benefit to patients [16,116,119]. Pain beyond the normal healing time or in the absence of any meaningful peripheral pathology has long been a challenge in the understanding of CNSLBP.

Sensitization of the nociceptive system is one suggested explanation for this problem. Enhanced synaptic efficiency of nociceptive networks may facilitate the perception of pain with little or no peripheral nociceptive input. Evidence indicates that there is some degree of sensitisation in CNSLBP patients [99,120,121]. Theoretically, this may be the result of changes in the periphery, the spinal cord [122,123], the brain [124] or in a combination of these areas.

Studies of inflammatory and neuropathic pain models in animals demonstrate extensive structural and functional alterations in the dorsal horn of the spinal cord that represent a relatively specific degeneration of the inhibitory interneurone system, leading to central sensitization and increases in pain-related behaviours (for review see [122]). Similar changes in the dorsal horn have been demonstrated post-mortem in human subjects who had suffered from post-herpetic neuralgia [125]. While it must be considered that CNSLBP develops in the absence of demonstrable peripheral nerve injury it is possible that the initial afferent nociceptive barrage that follows acute spinal injury may induce lasting alterations in dorsal horn function that lead to the maintenance of pain after tissue healing has occurred.

Methodological barriers to studying cord structure and function in humans make it difficult to ascertain the contribution of such changes to CNSLBP. Furthermore, in rodents, the spinal cord represents a far greater proportion of the CNS than in humans, and animal models demonstrate that while cord changes are prevalent in younger animals, brain changes dominate in older animals [126]. Nevertheless, the animal data is important as it suggests a common aetiology of neurodegeneration. While there is currently no evidence of spinal cord changes in CNSLBP patients, there is significant data on altered brain function [99,103,121,124,127]. It could be argued that supraspinal changes may be more likely to drive the sensitised state seen in human subjects with CNSLBP.

The work on brain changes in CLBP patients provides speculative support for this view. Schmidt-Wilke et al. [102] demonstrated gray matter degeneration of the brainstem, an area highly associated with inhibitory pain control, potentially leading to a loss of anti-nociception and the promotion of a sensitised state. There is also strong evidence for the role of the DLPFC in pain modulation and inhibition. Activity in the DLPFC has been shown to be negatively correlated with pain intensity and unpleasantness [128]. This area appears to have a role in affective disengagement from pain [129] and activity in this region is negatively correlated with the degree of pain related catastrophising [130]. These functions are likely to be affected by the extensive DLPFC degeneration seen in CLBP. In support of this idea, Baliki et al. [109] have shown that the sustained spontaneous pain of CLBP is strongly associated with increased activity of the medial pre-frontal cortex (mPFC) and suggest that this is the result of a loss of inhibitory control of the mPFC from the DLPFC.

A second proposition for the perception of pain in the absence of spinal pathology is the presence of central pain memories. Flor [131] has suggested that the alterations in sensory cortical representation found in chronic pain represent the neuronal substrate of implicit pain memories that serve to maintain and enhance pain perceived in the affected body region. As well as providing a mechanism for the maintenance of ongoing back pain, this may help to explain how previous episodes of low back pain lead to the development of chronicity, by contributing to and reinforcing these pain memories.

An alternative mechanism has been proposed by which cortical reorganisation may lead to the generation of ongoing, movement related pain from within the brain. Harris [132] suggests that altered cortical representation of somatic input may falsely signal incongruence between motor intention and movement. The generation of motor activity within the central nervous system is closely coupled to sensory feedback systems, which are monitored to detect any deviation from the predicted response [133]. If there is conflict between motor output and sensory feedback, it is hypothesised that pain is produced as a warning signal to alert the individual to abnormalities within information processing [133]. Alteration of the lumbar spine's representation in S1 [99] may be the substrate of abnormal proprioceptive representation of the back and therefore a possible source of sensory-motor incongruence.

McCabe et al. [133] provided some experimental support for this hypothesis by inducing pain in healthy volunteers moving in an environment that created a degree of sensory-motor conflict. These results have attracted some criticism [134] and research using an alternative methodology to disrupt cortical proprioceptive representation was unable to evoke pain [135]. It is worth considering that CNSLBP develops following an initial painful event and it is arguable that sensory-motor conflict may have more potential for generating pain when the pain 'matrix' is in a sensitised state.

Lack of visual input of the moving part is a critical component of the incongruence hypothesis [132]. Vision has a dominant role over other senses in self-recognition, and many studies demonstrate that body position sense is re-calibrated to conform to visual information (for review see Jeannerod [136]). Thus the lumbar spine, which is not directly visualised during task performance, may be more susceptible to this problem as abnormal cortical proprioceptive representation cannot be 'corrected' by visual feedback.

Interestingly, conditions that generate sensory-motor conflict are associated with increased activation in the right dorsolateral prefrontal cortex (DLPFC) [137]. As mentioned above structural neurodegeneration and altered activity of the DLPFC is a consistent finding in patients with CNSLBP [98,102,107], additionally a number of studies have shown these changes are localised to the right hemisphere [102,107]. Apkarian et al. [98] suggest excitotoxicity secondary to long-term potentiation as a possible cause of this degeneration. One possible candidate for inducing these changes is over-activation secondary to sensory-motor incongruence.

In summary, there are numerous ways whereby reorganisation within the brain may generate persistent pain in the absence of ongoing peripheral pathology. These include, reduced cortico-cortical and descending inhibition producing an abnormally sensitised state, the reactivation of implicit pain memories, and centrally generated pain in response to sensori-motor incongruence when the patient moves the back. It is plausible that these mechanisms coexist. It is currently unknown to what extent that these brain changes are reversible. Similar but distinct brain changes are noted in phantom limb pain and CRPS I, and both these conditions appear amenable to treatment directed towards the brain [110-114]. If some of the changes noted are not reversible it might still be possible to alter pain by affecting processing within this altered system.

### Biomechanical manifestations of CNSLBP

The biomechanical aspects of LBP have been widely investigated [138], and the range of mechanical manifestations seen in CNSLBP patients is vast. The back is moved less during functional tasks [139-143] and there is greater asymmetry and variability in performance [140,144,145]. There is a consistent decrease in the velocity of movement [146-148]. Muscles are activated in a dys-coordinated manner in static and dynamic situations [149,150]. When lifting, the back muscles are recruited earlier and stay on for longer and there is a greater amount of co-contraction [151-153]. There are altered patterns of muscle recruitment with limb movements [154], with sudden loading and unloading [155-158], and balance is impaired [157,159-162]. There is some suggestion of poorer proprioception [163-165] and there are delays in reaction times to a variety of tasks [166-168].

The congruence seen within these data is surprising. The overwhelming sense is a loss of confidence, control and coordination within the back. While Moseley and Hodges [169] contend that it is unclear if these abnormalities are compensatory, causative, neither or both, a common approach is to interpret these findings as causative. Poor stability and control is thought to influence loading of the lumbar spine and sustain the production of peripheral nociceptive input [35,154,170,171]. Within this framework treatment is directed towards normalising motor performance based on a peripheral biomechanical model. However, it may be that these physical abnormalities are not causative but result from altered higher centre representation and the individual's attempts to maintain function with an altered body schema. It is conceivable that these features are epiphenomena of cortical dysfunction rather than the underlying mechanism of CNSLBP. If this is the case treatment guided by a biomechanical explanatory model might be misdirected.

Support for this view can be seen in a number of areas. There appears to be little association between changes in patient centred outcome and changes in any aspect of low back related physical performance [117]. Lumbar stabilisation training programmes, which interpret these problems as causative and specifically address them from a biomechanical perspective [172] and other active [173] and passive [174] interventions aimed at changing the mechanical behaviour of the lower back demonstrate limited clinical effectiveness.

### Psychological manifestations of CNSLBP

Psychological factors are an important part of the chronic LBP experience. They contribute to the progression to chronicity, explain a significant amount of the variance among CLBP patients and have been identified as important barriers to resolution [44,175,176]. Alterations in brain function offer some additional insight into the role psychological features play in the development of CNSLBP and the maintenance of the problem once it is established.

Pincus et al. [175,176] cite distress/depressive mood as integral psychological factors in the transition from acute to chronic LBP and several psychosocial models have been postulated for the role of distress/depression in this transition [176,177]. Although speculative, brain reorganisation may also be significant. It appears that there is considerable overlap in the neural circuitries of the brain in both depression and CLBP [107]. It is of particular interest that alteration of DLPFC function has been strongly implicated in depressive disorders [178,179]. Although its precise role is currently not clearly defined [180,181], evidence points to primary abnormal activity within the DLPFC [107]. Depressed CNSLBP patients have been shown to have evidence of DLPFC degeneration and the level of degeneration was highly correlated with the level of depression [107]. One mechanism by which depressed mood influences chronicity may be via contributing to DLPFC degeneration.

Psychological variables also account for a significant amount of the variance in the CNSLBP condition [44]. Despite their explanatory value, the evidence for the efficacy of psychologically based treatments is not strong. Psychologically focused treatment only delivers small effects on pain and function when compared to no treatment [7]. They seem to be no more effective than other active interventions [7,117,182] and provide little additional benefit when added to other conservative treatment approaches [84,117,183]. The brain changes seen in CNSLBP may help explain this situation.

The fear-avoidance model is seen as a central psychological mechanism in the maintenance of CNSLBP [184,185]. Fundamental to this model are the opposing behavioural responses of confrontation and avoidance. Confrontation is seen as adaptive and likely to lead to resolution. Alternatively, avoidance is thought to be maladaptive and contribute powerfully to the development of chronicity and numerous associated biological and psychological sequelae [186]. Catastrophic beliefs and pain related fear are primarily what drives avoidance [184], and are consequently seen as the primary targets for intervention. The origins of these beliefs are viewed as a major challenge in developing the fear-avoidance model [186,187] and numerous possibilities have been suggested [184-187].

One possible candidate for the genesis of these beliefs is alteration in cortical representation of the back and resultant distorted body schema. Keefe et al [187] state that fear may be fuelled by unexpected and novel bodily sensations. Both McCabe et al. [133] and Moseley et al. [135] demonstrated that strangeness, foreignness and peculiarity are features of moving when there is sensory-motor incongruence. As with the biomechanical features of CNSLBP, fear and catastrophising may also be viewed as responses to an altered body schema. These beliefs would be a reasonable response to an individual with an altered cortical representation of their back who experiences unexplained pain and unexpected and novel sensations when moving. Treatments that seek to address these problems from a cognitive perspective may be less than ideal, as the fundamental substrate for the genesis of fear and catastrophisation would still be present.

It is also possible that degeneration within the DLPFC may contribute directly to greater catastrophic interpretation of pain. The DLPFC seems to be important in emotional disengagement from pain [128,129] and it has been proposed that degeneration of the DLPFC may contribute to the production of spontaneous pain [109], which may fuel catastrophic thoughts, and, as discussed above, there is evidence that DLPFC activity is negatively correlated with catastrophic thinking about pain [130].

Individuals' coping strategies are also considered to be important contributors to the disability associated with CNSLBP. Active coping strategies, characterised by efforts to function in spite of pain or to distract oneself from pain are thought to be important in adaptive functioning [188]. Psychological management of CNSLBP seeks to use distraction and encourages engagement in activity despite pain, as part of the treatment process [189]. This requires that the individual is able to let task relevant stimuli to dominate over pain [128], which can be particularly difficult given the high attentional demands of pain [190]. One suggested role of the DLPFC is in governing efficient performance in the presence of interfering stimuli [128]. Degeneration of the DLPFC will obviously interfere with this ability. Patients may not respond to this component of treatment as the neural substrate for engagement in this process is not available to them. These views do not challenge the biopsychosocial approach to the management of CNSLBP. It is abundantly clear that the thoughts, feelings and beliefs of an individual impact significantly on the problem [4,44,191]. What may require reconsideration is the origins of some maladaptive thoughts and beliefs and how these may be best modified.

**Figure 1:**
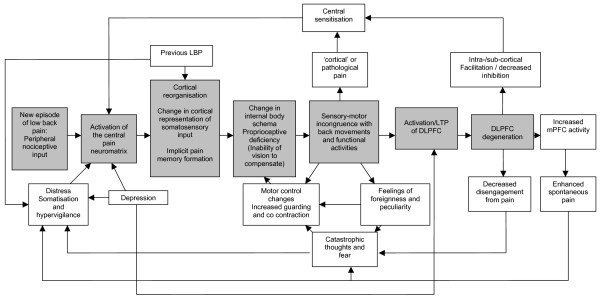
A Cortical Dysfunction Model of Chronic Non Specific Low Back Pain. Abbreviations: LTP = Long Term Potentiation, DLPFC = Dorsolateral Prefrontal Cortex, mPFC = medial Prefrontal Cortex.

Figure 1. presents a speculative model for the development and maintenance of NSCLBP with altered cortical function at its core. In the model, current and previous episodes of back pain contribute to an altered cortical representation of the back. Conceivably, previous episodes of LBP may also increase distress about the problem. Alterations in proprioceptive representation, subsequent sensory-motor incongruence and pre-existing depressive mood lead to over-activation and subsequent neurodegenerative change in the DLPFC. Sensory-motor incongruence may also directly produce pain, sustain altered motor control strategies and contribute to fear and catastrophic thoughts. The resulting DLPFC dysfunction contributes to central sensitization and subsequent ongoing and exaggerated pain, and also decreases the patient's ability to disengage from the pain, thus feeding back into the negative psychological influences. The mechanisms presented in the model should be acknowledged as speculative but represent an attempt to interpret the alterations in brain function that have been demonstrated in CNSLBP in light of the clinical manifestations of the condition.

In summary, CNSLBP patients have back pain yet no conservative or surgical pain relieving measures directed at the back appear effective. They display a number of biomechanical abnormalities, however treatment directed at normalising lumbar biomechanics has little effect and there is no relationship between changes in outcome and changes in spinal mechanics. Finally, these patients demonstrate some psychological problems but psychologically based treatments offer only partial solution to the problem. A possible explanation for these findings is that they are epiphenomena, features that are incidental to a problem of neurological reorganisation and degeneration.

### Equivalence of treatment effects

As discussed, the response of CNSLBP patients is very similar across a broad range of treatment options. A simple explanation would be to conclude that current interventions are ineffective or demonstrate only non-specific or placebo effects. However, the evidence does not support either of these perspectives. Most commonly applied conservative treatments demonstrate some level of effectiveness, albeit small, and this seems to be above what is produced by sham treatment [15]. Alternatively, disparate treatments may demonstrate equivalence in outcome because they all work through the same mechanism. Massage, manipulation, exercise, acupuncture, education and other interventions may work by influencing cortical function. The effectiveness is comparable across treatments because the pathway by which they act may be the same.

This perspective may also explain the disappointing effect sizes seen with conservative care. A possible reason for the small effects seen is that none of these interventions are currently delivered in a way that focuses on central processing. Mobilisation of the lumbar spine might be made more cortically directed by having the patient respond verbally to the stimulus, trying to localise the level or determine direction of pressure, akin to the sensory discrimination task employed by Flor et al. [114] in managing phantom limb pain or by Moseley et al. [192] in CRPS. Exercises aimed at improving trunk muscle recruitment seem an ideal way to normalise cortical representation, but rather than emphasis being placed on performing a particular type of muscle recruitment pattern based on a peripheral biomechanical model [154,170,171] participation in a variety of challenging exercises may be more useful. Simply mastering any skill that the patient finds difficult may be all that is required.

Similar thinking can be applied to most commonly applied conservative interventions, and whilst changing the emphasis of existing treatments may be a viable option for enhancing clinical efficacy, it may also be useful to consider other possibilities. There is mounting evidence for the effectiveness of alternative and innovative strategies for "re-training" cortical function in other complex, long standing, pain problems [110-114,193] as well as recent data that training of brain activation with functional MRI can favourably influence chronic pain [194]. Advances in non-invasive brain stimulation techniques such as repetitive transcranial magnetic stimulation and transcranial direct current stimulation also offer approaches for altering cortical function to achieve pain relief [195,196] and to enhance cognitive function [197-199]. The challenge presented by this model for researchers and clinicians is to determine how therapy might be designed to address cortical function.

In conclusion there is little evidence to endorse current treatment for CNSLBP. Most treatments provide only small short-term changes and there is currently scant evidence that one form of treatment is superior to another. Sub-grouping of CNSLBP is a common response to these disappointing results. The sub-grouping perspective is not consistently supported by the available data and it is important that other research agendas are entertained. An alternative perspective is that treatment has been ineffective because it has been misdirected. CNSLBP may be a problem of abnormal cortical function. There is growing evidence of supraspinal abnormalities in CNSLBP, these mechanisms can potentially explain the complexity of the LBP experience and the outcomes of clinical trials on CNSLBP. Various research groups have demonstrated the potential value of 'training the brain' in other conditions characterised by abnormal cortical processing. Given the growing body of evidence indicating similar neural mechanisms in CNSLBP, the development of clinical strategies targeted at normalising neurological processing represents a challenging new direction for musculoskeletal clinicians and researchers involved in the management of CNSLBP.

## Summary

• Clinical trials offer little support for current management of chronic non specific low back pain

• Sub-grouping has been commonly offered as an explanation for the disappointing results of clinical trials

• Much of the literature on CNSLBP do not strongly support a sub-grouping model

• Evidence is emerging of significant cortical alterations in CNSLBP patients

These changes may offer an explanation for the complex problem of CLBP and a potential focus for effective treatment.

## Competing interests

The author(s) declare that they have no competing interests.

## Authors' contributions

Both authors contributed to the development of the ideas and arguments within this manuscript. Both authors have read and approved the manuscript

## Pre-publication history

The pre-publication history for this paper can be accessed here:


